# Loss of complex O-glycosylation impairs exocrine pancreatic function and induces MODY8-like diabetes in mice

**DOI:** 10.1038/s12276-018-0157-3

**Published:** 2018-10-10

**Authors:** Gerrit Wolters-Eisfeld, Baris Mercanoglu, Bianca T. Hofmann, Thomas Wolpers, Claudia Schnabel, Sönke Harder, Pascal Steffen, Kai Bachmann, Babett Steglich, Jörg Schrader, Nicola Gagliani, Hartmut Schlüter, Cenap Güngör, Jakob R. Izbicki, Christoph Wagener, Maximilian Bockhorn

**Affiliations:** 1 Department of General, Visceral and Thoracic Surgery, University Medical Center Hamburg- Eppendorf (UKE), Hamburg, Germany; 2 Metabolic Laboratory and Newborn Screening, University Medical Center Hamburg-Eppendorf (UKE), Hamburg, Germany; 3 Mass Spectrometric Proteomics—Institute for Clinical Chemistry & Laboratory Medicine, University Medical Center Hamburg-Eppendorf (UKE), Hamburg, Germany; 4 Center for Diagnostics, University Medical Center Hamburg-Eppendorf (UKE), Hamburg, Germany

## Abstract

Cosmc is ubiquitously expressed and acts as a specific molecular chaperone assisting the folding and stability of core 1 synthase. Thus, it plays a crucial role in the biosynthesis of O-linked glycosylation of proteins. Here, we show that ablation of Cosmc in the exocrine pancreas of mice causes expression of truncated O-glycans (Tn antigen), resulting in exocrine pancreatic insufficiency with decreased activities of digestive enzymes and diabetes. To understand the molecular causes of the pleiotropic phenotype, we used *Vicia villosa* agglutinin to enrich Tn antigen-modified proteins from Cosmc-KO pancreatic lysates and performed a proteomic analysis. Interestingly, a variety of proteins were identified, of which bile salt-activated lipase (also denoted carboxyl-ester lipase, Cel) was the most abundant. In humans, frameshift mutations in CEL cause maturity-onset diabetes of the young type 8 (MODY8), a monogenic syndrome of diabetes and pancreatic exocrine dysfunction. Here, we provide data suggesting that differentially O-glycosylated Cel could negatively affect beta cell function. Taken together, our findings demonstrate the importance of correct O-glycan formation for normal exocrine and endocrine pancreatic function, implying that aberrant O-glycans might be relevant for pathogenic mechanisms of the pancreas.

## Pancreatic disorders: when protein modification misfires

Reduced levels of a protein called Cosmc in the pancreas may lead to pancreatic disorders and a rare form of diabetes. Disruption to glycosylation, the process by which carbohydrates are added to proteins, can have significant knock-on effects for cellular functioning. Gerrit Wolters-Eisfeld at the University Medical Center Hamburg-Eppendorf, Germany, and co-workers used mouse models to demonstrate the importance of correct formation of one type of carbohydrate, O-glycans, for normal pancreatic function. The team generated mice without Cosmc, a molecule that assists with O-glycosylation. Loss of Cosmc in the pancreas resulted in shortened O-glycans and pancreatic insufficiency. However, one protein called Cel was modified in abundance; this may be further bad news because mutations in Cel are linked to the onset of a rare form of diabetes.

## Introduction

**Fig. 1:**
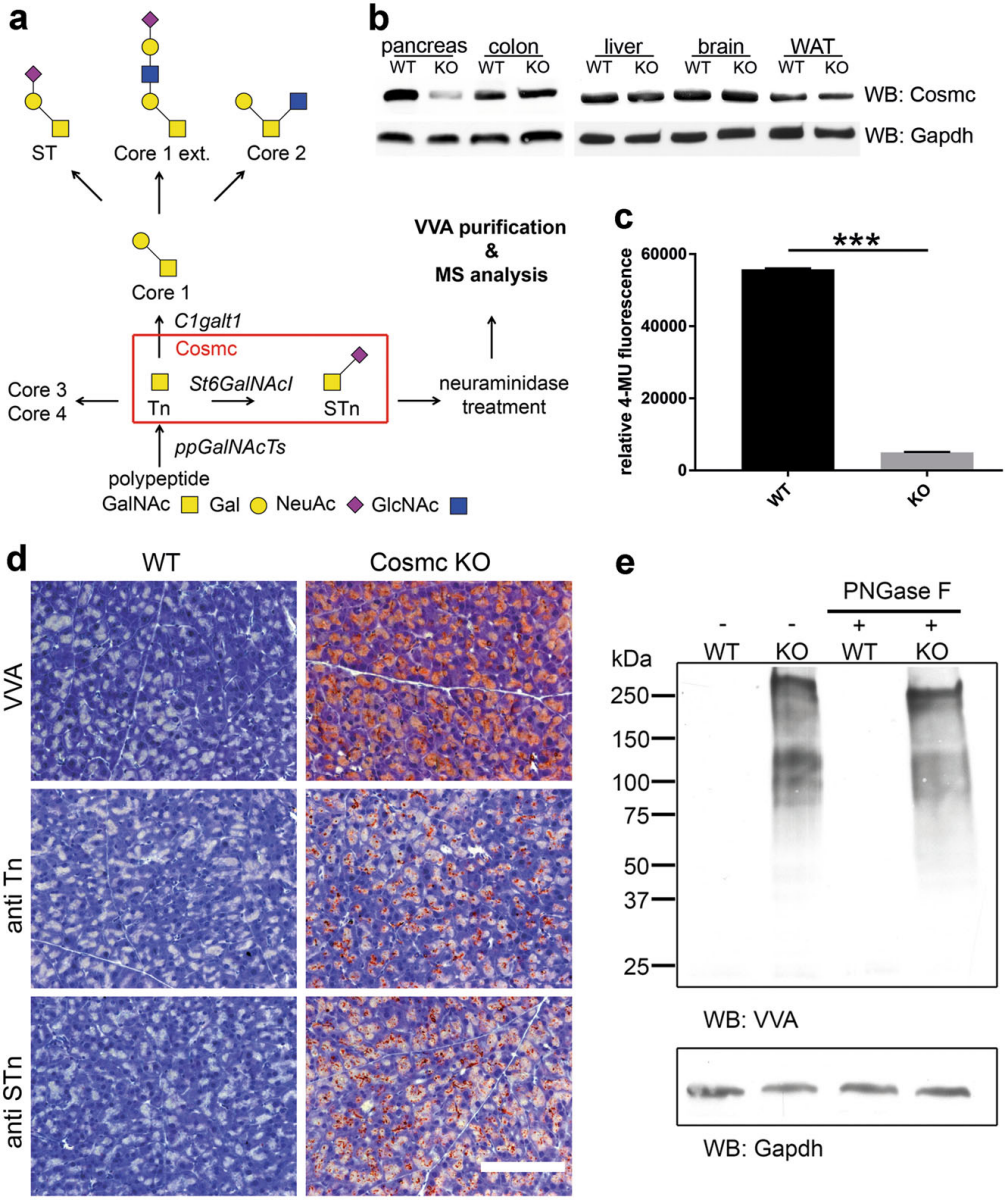
Deletion of *Cosmc* results in loss of core 1 glycan formation. **a** Scheme of O-glycosylation pinpointing the role of Cosmc. Depending on the biological context, a set of expressed ppGalNAcTs transfer GalNAc moieties to serine and threonine residues of the target proteins. Subsequent extension from Tn antigen to core 1 is performed by C1galt1 (T-synthase) being folded and stabilized by Cosmc. Lack of T-synthase activity results in Tn and STn expression. **b** Anti-Cosmc Western blot analysis of pancreas, colon, liver, brain and white adipose tissue (WAT) lysates derived from WT and pancreas-specific *Cosmc-*KO mice. Clearly reduced Cosmc protein is observed in KO pancreatic tissue. **c** The fluorescent T-synthase assay further validated reduced T-synthase activity in *Cosmc-*KO pancreatic tissue compared to WT (*n* = 4). **d** Representative histochemical staining using VVA on FFPE WT and *Cosmc-*deficient mouse pancreata demonstrates specific binding to acinar cells in *Cosmc* KO. Further, antibodies directed against Tn and STn antigens specifically detected pancreatic acinar cells in *Cosmc* KO. The scale bar equals 50 µm. **e** Far-Western blot analysis of whole-pancreatic tissue lysates of *Cosmc-*KO mice display Tn antigen expression on a variety of proteins, whereas no reactivity is observed in lysates of WT littermates. PNGase F treatment did not alter VVA reactivity in *Cosmc-*KO lysates, emphasizing VVA specificity towards O-linked GalNAc

Among the multifarious spectrum of post-translational modifications, glycosylation is the most common and most diverse one. For some forms of glycosylation, there is substantial knowledge of their synthesis and biological functions. However, our knowledge of O-linked glycosylation is severely limited and represents an emerging field in research^1^. O-glycans are covalently α-linked via an *N*- acetylgalactosamine (GalNAc) moiety to Ser and Thr residues^2,3^ by the enzyme family of polypeptide N-acetylgalactosaminyltransferases (ppGalNAcTs) to generate the O-glycan precursor, which is also called Tn antigen (Tn)^4^. The most common modification of Tn is the formation of the core 1 (T antigen) disaccharide catalyzed by T-synthase^5^. The activity of T-synthase requires its chaperone C1galt1c1 (hereafter Cosmc)^6^, which mediates proper protein folding in the endoplasmic reticulum (Fig. 1a)^7^.

The mammalian pancreas is a glandular organ with endocrine and exocrine functions. While endocrine cells are located in the islets of Langerhans and secrete insulin, glucagon, somatostatin and pancreatic polypeptide, the exocrine acinar cells produce inactive precursors of digestive enzymes that are stored in zymogen granules. After stimulation, zymogen granules migrate to the apical plasma membrane and release their contents into pancreatic ducts that drain into the duodenum. Glycans participate in many key biological processes, including cell adhesion, molecular trafficking and clearance, receptor activation, signal transduction, and endocytosis^8^. On this basis, the role of Tn and its impact on pancreas physiology and disease was be investigated in this study. We generated a conditional floxed *Cosmc* mouse strain that was interbred with a *pancreas-specific transcription factor 1a* (Ptf1a) Cre strain to obtain pancreatic *Cosmc-*KO offspring. Characterization of *Cosmc*-deficient mice revealed symptoms of exocrine pancreatic insufficiency with maldigestion, impaired zymogen granule release and decreased enzymatic elastase and lipase activity. Fasting serum glucose and glycated hemoglobin (HbA1c) were elevated, and fasting serum C-peptide was reduced. Of note, cells of the endocrine pancreas were not affected by conditional *Ptf1a*-driven^9^ *Cosmc* ablation, suggesting molecular crosstalk between exocrine and endocrine cell populations^10^.

*Cosmc* deficiency and consecutive Tn antigen expression in the exocrine pancreas enables O-GalNAc glycoprotein identification using the plant lectin *Vicia villosa* agglutinin (VVA)^11^ for purification and subsequent mass-spectrometric proteome analysis^12^. It is assumed that differential O-glycosylation acts as the sum of all proteins involved, making Cosmc a powerful regulatory switch.

Carboxyl ester lipase, also known as bile salt-activated lipase, is highly expressed in pancreatic acinar cells. This digestive enzyme is activated by bile salts in the duodenum and participates in the hydrolysis and absorption of cholesterol and lipid-soluble vitamins^13^. The last of the 11 *CEL* exons contains a variable number of tandem repeats (VNTR) region. Single-base deletions in the *CEL* VNTR cause an autosomal, dominantly inherited pancreatic disease characterized by diabetes and exocrine dysfunction, named maturity-onset diabetes of the young type 8 (MODY8)^14^. CEL is heavily O-glycosylated at sites located in the VNTR at the protein C-terminus^15^. The VNTR is not required for functional properties of the enzyme, such as catalytic activity and activation by bile salts^16^. However, O-glycosylation has been suggested to be necessary for proper folding, secretion and stability^17^. Our data indicate that loss of core 1-derived O-glycans on Cel has effects analogous to nonsense point mutations causing MODY8-like diabetes. This study demonstrates the impact of differential O-glycosylation on pancreas function and underscores the relevance of O-glycosylation to pathophysiological mechanisms in vivo.

## Materials and methods

### Generation of pancreatic acinar cell-specific Cosmc-knockout mice

Based on the *Cosmc* cDNA sequence NM_021550 a targeting strategy was chosen, leading to the conditional deletion of *Cosmc* exon 2 by insertion of a lox site in intron 1 and a second lox site combined with an FRT-flanked neomycin selection cassette downstream of exon 2. This strategy resulted in the conditional deletion of the entire coding sequence. Male chimeras were bred, and heterozygous mice were generated. The conditional *Cosmc*-knockout model was generated by genOway (Lyon, France). *Cosmc*^fl/fl^ females were crossed with *Ptf1a*^cre/+ ^;C*osmc*^−/y^ males to generate homozygous *Cosmc*-KO and wild-type littermates. Homozygous Cosmc-KO male and female mice displayed the same phenotype. All mice were on a C57BL/6 J background. Genotyping was performed using the Kappa mouse genotyping hot start Kit (PeqLab, Erlangen, Germany). Genotyping primers were Cre-F 5′-ACCAGCCAGCTATCAACTCG-3, Cre-R 5-TTACATTGGTCCAGCCACC-3′, *Cosmc*-F 5′- CACAGAACTCACTATCCACTAGGCATGAATACAT-3′; *Cosmc*-R 5′-GCTCTCCCTAAATATACAACCGATTAAGAAAGTGT-3′. All animal experiments were performed in accordance with the National Institutes of Health Guide for the Care and Use of Laboratory Animals and were approved by the local animal ethics committee.

### Isolation and primary culture of islets and acinar cells

Fresh pancreata were perfused with 3–5 ml of collagenase (Sigma-Aldrich, St. Louis, MO, USA) solution in 0.5 mg/ml HBSS on ice and incubated for 20 min at 5% O_2_ and 37 °C^18^. Cells were carefully separated by pipetting up and down and washed with HBSS by centrifugation (500 g for 2 min). Resuspended cells were poured through a 1000-µm mesh, washed again and resuspended in 25% Ficoll solution. A Ficoll gradient was prepared by layering 2.4 ml of each of 23, 20.5 and 11% Ficoll and centrifuging at 800 g for 15 min. Islets of Langerhans were pelleted and washed in HBSS. Lysis was performed using RIPA buffer. Genomic DNA (gDNA) was purified using DNeasy Blood & Tissue Kit (Qiagen, Hilden, Germany). Acinar cells were prepared and cultured at 1 × 10^6^/ml in 24-well plates and allowed to attach for 24 h^19^. The cells were treated with 10^–8^ M cerulein (Sigma-Aldrich) for 2 h. Cell culture supernatants were analyzed for levels of lipase and elastase.

### Isolation of pancreatic zymogen granules

For isolation of pancreatic zymogen granules, pancreata were homogenized in homogenization buffer (0.3 M sucrose, 2 mM MOPS, pH 6.8, Halt protease inhibitors, 1 mM EDTA) and centrifuged at 750 g for 10 min to remove cell debris and nuclei. Supernatants were filtered through 20-µm nylon mesh and flow-through-centrifuged at 1750 g for 10 min to pellet zymogen granules^20^. Granules were lysed using RIPA buffer.

### Western blot, IHC/IF, VVA pulldown and real-time PCR

These experiments were performed as we previously described^21–23^. For Western blot, antibodies for GAPDH (sc-32233; Santa Cruz Biotechnology, Santa Cruz, CA, USA), Cosmc (sc-67480; Santa Cruz Biotechnology), CEL (ab79131; Abcam, Cambridge, UK; sc-34883; Santa Cruz Biotechnology), DMBT1 (MAB59151; R&D Systems, Minneapolis, MN, USA), elastase (ab21593; Abcam) and trypsin (ab166898; Abcam) were used. Ten micrograms per milliliter of *Vicia villosa* lectin (VVA) reactive against O-GalNAc (Tn antigen) (B-1235; Vector Laboratories, Burlingame, CA, USA) complexed with 1 µg streptavidin-HRP (21126; Pierce, Thermo Fisher Scientific, Grand Island, NY, USA) was used. For immunohistochemistry, antibodies for Tn Antigen (MA180055; Thermo Fisher Scientific, Grand Island, NY, USA), STn Antigen (ab115957; Abcam), insulin (8138; CST, Beverly, MA, USA) and VVA-fluorescein (FL-1231; Vector Laboratories) were used at a dilution of 1:100^23^. Ten micrograms per milliliter of *Sambucus nigra* lectin (SNA) (B- 1305–2; Vector Laboratories, Burlingame, CA, USA) was complexed with 1 µg streptavidin-HRP. For real-time PCR, RNA was extracted with the RNeasy Plus Tissue Mini Kit (Qiagen).

### Enzyme-linked immunosorbent assay (ELISA)

All ELISAs were performed as proposed by the manufacturer (Cusabio, College Park, MD, USA). Mice were fasted for 4 h prior to blood collection. Mouse C-peptide was measured from undiluted plasma. Mouse glycated hemoglobin A1c (HbA1c) was measured from red blood cell lysates. Lipase and elastase were measured from total lysates and isolated zymogen granules (1 µg total protein).

### Statistical analysis

All data are expressed as the mean ± SEM. Statistical analysis was performed with Prism 7.03 software (GraphPad Software, La Jolla, CA, USA). All data were tested for normality by applying the D’Agostino-Pearson omnibus test. If normality was confirmed and there were no significant differences in variance between groups, the two-tailed Student *t* test was used to evaluate the differences.

### Quantification of islets

IF staining for insulin was performed for 5 WT and 5 Cosmc-deficient pancreata. Insulin-positive areas were measured with Keyence image analysis tool. For quantitation of Islets, ratios of islet area/total section area were log-transformed and analyzed by the Wilcoxon test. *P* < 0.05 denoted a statistically significant difference.

### Biochemistry analyses

Blood samples were centrifuged at 13,000 rpm for 10 min in a Biofuge 13 (Haereus, Hanau, Germany), and the analytes were determined from undiluted or 1:2-diluted serum (cholesterol, triglycerides, high-density lipoproteins). Samples were diluted with distilled water and were measured in a Dimension Vista 1500 (Siemens, Bad Nauheim, Germany). Analytes were measured by endpoint assays.

### Recombinant protein expression

Recombinant 6xHIS tagged CEL and CEL-Tn (Clone ID: OHu22073C) was expressed in HEK293 and COSMC-KO HEK293-SC (simple cells)^3^ and purified from Pro293 a Medium (Lonza, Basel, Switzerland) using Ni-NTA agarose (Qiagen).

### NT-3 insulin measurement and viability assay

The cell line NT-3 was generated from a surgically resected lymph node of a 33-year-old male patient with a well-differentiated neuroendocrine tumor of the pancreas^24^. Human insulin content was determined using the ADVIA Centaur Insulin Assay (REF 02230141) and ADVIA Centaur XP analyzer (Siemens Healthcare, Munich, Germany). This assay is standardized to the first human insulin international reference preparation by the World Health Organization (NIBSC code 66/304). Cell culture supernatants of NT-3 cells were analyzed after stimulation with 25 mM Glucose for 60 min in the presence of 20 µM recombinant CEL or CEL-Tn. Cell viability was assessed using the CellTiter-Glo luminescent cell viability assay (Promega, Madison, WI, USA) after 48 h of 20 µM CEL or CEL-Tn treatment.

### Protein identification by mass spectrometry

Tn modified proteins were identified by LC-MS/MS. Briefly, the enriched Tn-modified proteins were in-gel purified and excised from the polyacrylamide gel. MS/MS spectra obtained by collision-induced fragmentation after manual precursor selection were evaluated by the Mascot MS/MS search algorithm version 2.3 (Matrix Sciences, Boston, MA, USA) or SEQUEST HT.

### T-synthase and endogenous enzyme assays

Ten micrograms of total protein from fresh tissue lysates was directly used to determine T-synthase activity using UDP-Gal (Sigma-Aldrich) as the donor and GalNAc-α-4-methylumbelliferyl (GalNAc-α-4-MU) (Carbosynth, Compton, UK) as the acceptor^25^. O-Glycosidase (New England Biolabs, Frankfurt am Main, Germany) was used for T antigen cleavage and release of fluorescent 4-MU. Fluorescence was measured (ex 355 nm/em 460 nm) in black OptiPlate-96 F plates (PerkinElmer, Waltham, MA, USA) in a FLUOstar Omega (BMG Labtech, Ortenberg, Germany). One microgram of total protein from fresh tissue lysates was directly used to determine lipase activity using 4-methylumbelliferyl butyrate (19362, Sigma-Aldrich) or pancreatic elastase using N-methoxysuccinyl-Ala-Ala-Pro-Val-7-amido-4-methylcoumarin (M9771, Sigma-Aldrich) as substrate. Fluorescence was measured (ex 355 nm/em 460 nm) after 30 min.

## Results

To investigate the role of O-glycosylation in the function of the pancreas in vivo, we generated a conditional *Cosmc* flox mouse line that was interbred with a *pancreas specific transcription factor 1a* (*Ptf1a*)-Cre line to induce Tn antigen expression in pancreatic acinar cells (supplemental Fig. 1). A PCR-based genotyping protocol was established to identify wild-type (*Cosmc* WT) and activated hetero- (*Ptf1a*^+/Cre^;*Cosmc*^+/fl^) and homozygous *Cosmc*-deficient (*Ptf1a*^+/Cre^;*Cosmc*^fl/fl^) animals, hereafter called *Cosmc*-KO (supplemental Fig. 2).

To verify the ablation of *Cosmc* on the mRNA and protein levels and the resulting enzymatic T-synthase inactivity, we first probed whole pancreatic lysates with Cosmc-specific antibody. We observed a distinct decrease in Cosmc protein in *Cosmc*-KO-derived pancreas compared to WT, while in colon, liver, brain and white adipose tissue no difference was detectable (Fig. 1b). Corresponding results were obtained when *Cosmc* mRNA expression in the pancreas was measured by quantitative RT-PCR (supplemental Fig. 3). Compared to WT, *Cosmc* mRNA was reduced by greater than 80% in *Cosmc*-KO mice. Interestingly, we found that the *T-synthase* gene was overexpressed in the pancreas of *Cosmc-*KO mice, suggesting a possible regulative feedback mechanism (supplemental Fig. 3). Ultimately, we tested T-synthase activity in pancreatic lysates derived from WT and *Cosmc-*KO mice. The results clearly indicate dysfunctional T-synthase in *Cosmc*-KO mice compared to WT (Fig. 1c).

In proof-of-concept experiments, we further subjected pancreata of WT and *Cosmc*-KO mice to histological staining using monoclonal antibodies and specific lectins directed against Tn and STn (Fig. 1d). Immunohistochemical staining using streptavidin-horseradish peroxidase complexed with biotinylated VVA on formalin-fixed, paraffin-embedded WT and *Cosmc-*KO mouse pancreata demonstrated specific binding of VVA in exocrine glandular cells of *Cosmc-*KO mice. Further, antibodies directed against Tn and STn antigens specifically bound to *Cosmc-*KO pancreatic acinar cells, indicating that a relevant portion of expressed Tn antigen was sialylated.

Immunofluorescence using fluorescein-VVA confirmed specific binding to pancreatic acinar cells in *Cosmc-*KO tissue (supplemental Fig. 4). Western and far-Western blot analysis of whole-pancreatic tissue lysates of *Cosmc-*KO mice displayed Tn antigen expression on a variety of proteins, whereas no reactivity was observed in lysates from WT littermates (Fig. 1e). We additionally used biotinylated lectin HPA and anti-Tn antigen antibody for Tn antigen detection on O-GalNAc-modified glycoproteins on *Cosmc-*KO pancreatic acinar cells (supplemental Fig. 5). In addition, WT and *Cosmc-*KO pancreatic tissue lysates were treated with N-glycosidase F (PNGase F) to remove N-glycans. PNGase F treatment did not alter VVA reactivity in *Cosmc-*KO lysates (Fig. 1e).

**Fig. 2:**
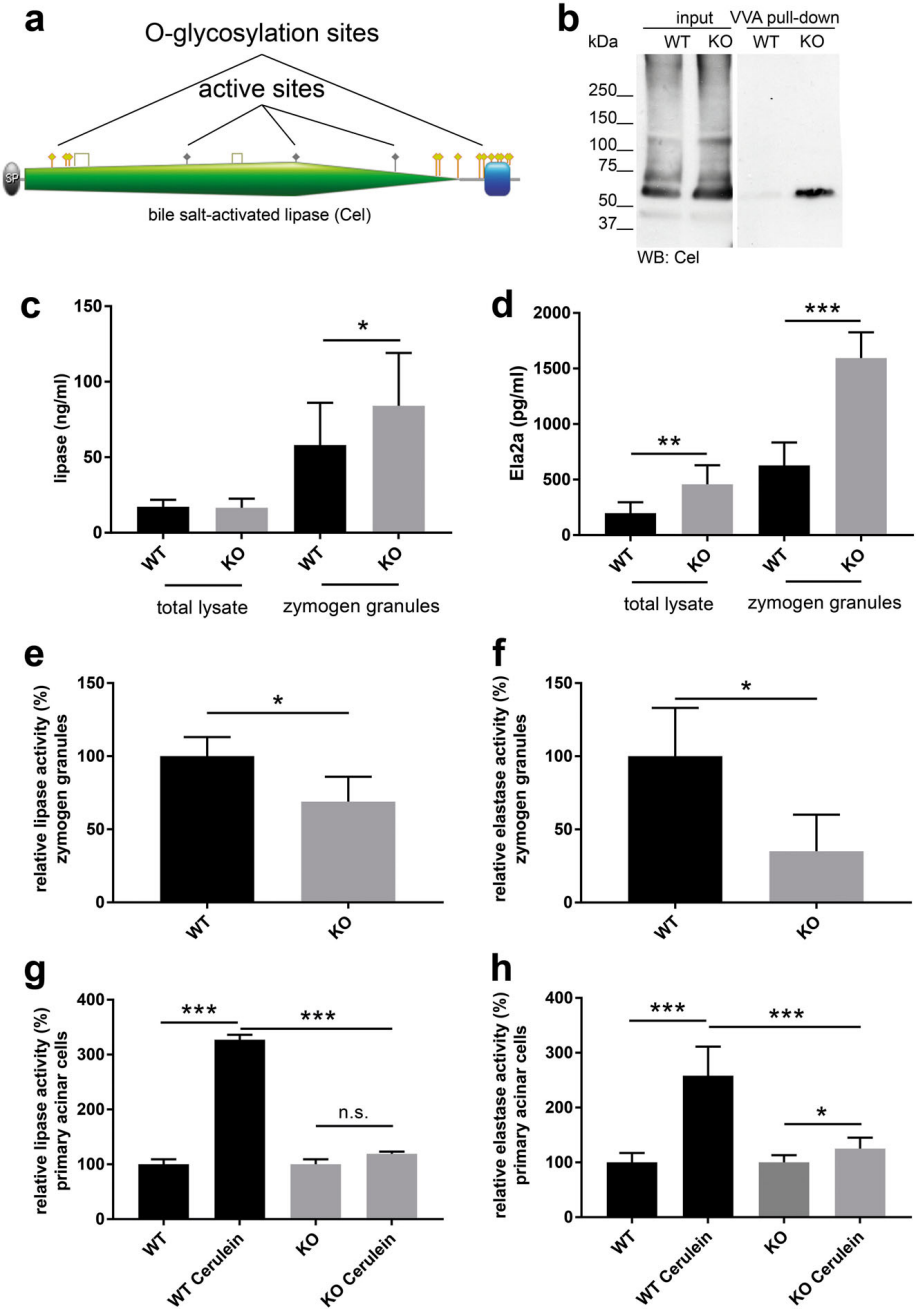
Cosmc-dependent O-GalNAc glycosylation affects pancreatic lipase and elastase enzyme function as well as exocrine pancreas secretion. **a** Scheme of bile salt-activated lipase (Cel) protein domains (SP, signal peptide; carboxylesterase, type B, green; PEST domain, blue; O-GalNAc glycosylation sites, yellow; active sites, gray). **b** Representative WB of VVA pull-down from WT and *Cosmc-*KO pancreatic lysates and Cel-specific antibody for detection. *Note*: Distinct Cel protein bands were robustly detected in pull-downs from KO tissue. **c** Absolute quantification of lipase in total lysates and zymogen granule lysates from WT and *Cosmc-*KO mice (*n* = 18). Total lysates display equal amounts of lipase (*P* = 0.748), whereas elevated levels are detected in *Cosmc-*KO zymogen granules (*P* = 0.023). **d** Absolute quantification of elastase (Ela2a) in total and zymogen granule lysates from WT and *Cosmc-*KO mice (*n* = 18). Lysates derived from Cosmc KO display significantly elevated elastase. (**e** + **f**) Relative lipase and elastase activities in zymogen granule lysates from WT and *Cosmc-*KO mice (*n* = 4) using 4-MU substrates reveal decreased activities in KO. (**g** + **h**) Analysis of lipase and elastase activities in response to cerulein stimulation of WT and *Cosmc-*KO primary pancreatic acinar cells. Cerulein-induced lipase and elastase secretion is detectable in WT cells (*P* = 0.0001) and is attenuated in *Cosmc-*KO mice (*P* = 0.611 and *P* = 0.04). Relative difference in stimulated lipase activity is *P* = 0.0001

**Fig. 3:**
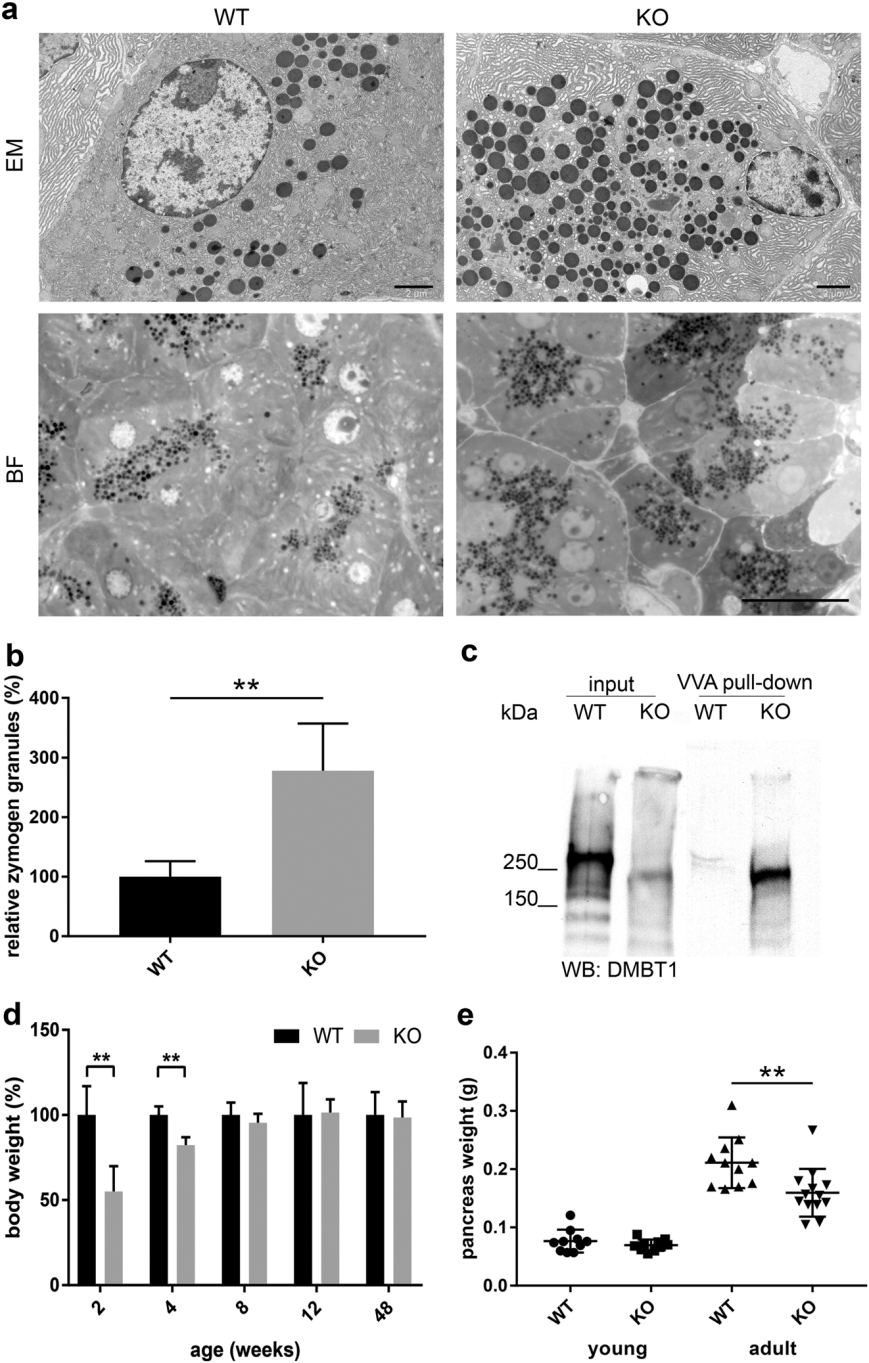
*Cosmc* deficiency results in accumulation of zymogen granules and pancreas atrophy in aged mice. **a** Representative TEM and brightfield micrographs of pancreas sections from fasting WT and *Cosmc*-KO adult mice. Zymogen granules are depicted as electron dense vesicles. TEM and BF scale bars are 2 and 20 µm, respectively. **b** Quantification of zymogen granules in five randomly selected fields of pancreas sections from fasting WT and *Cosmc-*KO adult mice (*n* = 5 each). Elevated numbers of zymogen granules in *Cosmc-*KO pancreata (*P* = 0.0004). **c** WB of VVA pull-down from WT and *Cosmc-*KO pancreatic lysates and DMBT1-specific antibody for detection. **d** Body weight analysis of WT and *Cosmc-*KO mice at ages 2–48 weeks (*n* = 10 each) displays reduced body weight gain at 2 and 4 weeks of age (*P* = 0.0021 and *P* = 0.0002). **e** Assessment of pancreas organ weight of 20- (*n* = 10) and 54-week-old WT (*n* = 11) and *Cosmc-*KO (*n* = 13) mice. Aged mice display reduced organ weight (*P* = 0.0062)

We next employed VVA-based enrichment of Tn-modified glycoproteins with subsequent LC-MS/MS analysis. Cel is one of the highly abundant, differentially O-glycosylated proteins expressed in the murine exocrine pancreas (Table 1). Cel belongs to the type-B carboxylesterase/lipase family and possesses a C-terminal domain with VNTR, a PEST motif and a high proportion of O-glycan moieties (Fig. 2a)^14^. Western blot analysis of pancreatic lysates using a Cel antibody showed bands at approximately 65, 75 and 120 kDa. VVA pull-down and subsequent detection with the same Cel antibody displayed a prominent band specifically in the *Cosmc-*KO sample at 65 kDa (Fig. 2b). Further experimentally validated Tn-modified proteins are shown in Fig. 3c and supplemental Figure 6.

**Table 1:** Top 10 O-GalNac-modified proteins identified by VVA lectin precipitation coupled with LC-MS/MS proteome analysis. The complete table is available in the supplement

Accession	Protein	Gene	Score
Q64285	Bile salt-activated lipase	Cel	516.25
Q6P8U6	Pancreatic triacylglycerol lipase	Pnlip	471.75
P10126	Elongation factor 1-alpha 1	Ef1a1	464.96
P08113	78 kDa glucose-regulated protein	Grp78	444.59
P20029	Endoplasmin	Enpl	360.26
P00688	Pancreatic alpha-amylase	Amy2	325.07
Q9DBG6	Dolichyl-diphosphooligosaccharide--protein glycosyltransferase subunit 2	Rpn2	296.01
Q8VDJ3	Vigilin	Hdlbp	285.34
Q9JKR6	Hypoxia upregulated protein 1	Hyou1	233.90
O88986	2-Amino-3-ketobutyrate coenzyme A ligase, mitochondrial	Gcat	224.19

To better understand the role of O-glycosylation on exocrine pancreatic function, we quantitated protein levels of pancreatic lipase and elastase (Ela2a) in total lysates and zymogen granules using ELISA. Pancreatic lipase had similar expression levels between WT and *Cosmc-*KO in total lysates but was elevated in *Cosmc-*KO zymogen granules (Fig. 2c). Elastase was significantly increased in both total lysates and zymogen granules of *Cosmc* KO (Fig. 2d). Analysis of relative lipase and elastase activities in zymogen granules normalized to actual lipase and elastase protein content revealed significantly reduced activities in *Cosmc-*deficient mice (Figs. 2e, f), indicating a direct impact of aberrant O-GalNAc glycosylation on enzymatic activity/activation. To evaluate the impact of core-1 derived O-glycans on physiological exocrine potential, primary pancreatic acinar cells derived from fasted WT and *Cosmc-*KO animals were prepared, cultured and cerulein stimulated. Cerulein is a gastroregulatory molecule with actions similar to cholecystokinin and thus induces the release of digestive enzymes from the pancreas. Subsequently, lipase and elastase activities from cell culture supernatants were measured using a specific 4-MU substrates (Figs. 2g, h). The stimulatory response to cerulein in WT acinar cells was apparent, whereas a significant reduction was obvious in *Cosmc-*KO cells.

To analyze the possible impact of aberrant O-glycosylation on ER function as well as zymogen granule formation and maturation, adult and fasting WT and *Cosmc-*KO pancreata (*n* = 3) were subjected to transmission electron microscopic analysis (Fig. 3a). No gross abnormalities were obvious, and the ER had a normal structure, indicating no ER stress induced by protein misfolding and/or ER-associated degradation^26^. However, the number of zymogen granules in *Cosmc-*deficient pancreata was considerably increased. Systematic counting of electron-dense zymogen granules confirmed a significant three-fold elevation in *Cosmc-*KO mice compared to WT (Fig. 3b). Among the identified Tn antigen-modified glycoproteins, we found the known heavily N- and O-glycosylated mucin-like protein DMBT1 (deleted in malignant brain tumors 1). DMBT1 has been described as a structural zymogen granule protein involved in the regulation of digestive enzyme secretion. It was subjected to Western blot analysis, which revealed a notable difference in the apparent DMBT1 molecular weight between WT and *Cosmc* KO. The down-shift in KO lysates clearly underscores the loss of complex O-glycosylation. For validation, VVA pull-down discriminated WT from *Cosmc-*KO DMBT1 glycoforms (Fig. 3c).

Exploration of the small and large intestine of non-fasting WT mice revealed semisolid stool in the distal small intestine and fully formed pellets of stool in the proximal colon. In contrast, *Cosmc-*KO mice displayed a brightened, semisolid stool in the distal colon, indicating insufficient digestion and malabsorption. A laboratory analysis of blood samples from WT and *Cosmc-*KO animals revealed differences in lipid profiles related to exocrine pancreatic function, with decreased total cholesterol, triglycerides and high-density lipoprotein (HDL) in *Cosmc-*KO animals (Table 2).

**Table 2:** Laboratory values of WT and *Cosmc-*KO mouse blood samples (*n* = 5 each). Unpaired two-tailed Student’s *t*-test was used to test the level of significance

Characteristic	Wild-type (mean ± SD)	Cosmc KO (mean ± SD)	*P* value	95% CI
Total cholesterol (mg/dl)	129.3 ± 11.7	103.2 ± 7.1	0.007	9.95–42.05
Triglycerides (mg/dl)	110.0 ± 21.6	94.4 ± 10.8	0.211	−11.69–42.89
High-density lipoprotein (mg/dl)	152.0 ± 4.0	96.4 ± 15.4	0.001	36.61–82.58

Furthermore, we monitored body weights of male WT and *Cosmc-*KO littermates at 2, 4, 8, 12, 24 and 48 weeks of age. A significant decrease in body weight gain in young (2 and 4-week-old) *Cosmc-*KO animals was observed. This phenotype was reversed at 8 weeks of age and was no longer observable at later time points (Fig. 3d). The *Cosmc-*KO pups did not exhibit signs of negligence, abnormal coat state or poor breast-feeding behavior. Pathological assessment of hematoxylin and eosin (HE)-stained WT and *Cosmc-*KO pancreatic sections revealed that pancreatic organ development was unaltered, and no gross abnormalities were observed. However, 1-year-old *Cosmc-*KO mice displayed pancreatic atrophy (Fig. 3e and supplemental Figure 7).

**Fig. 4:**
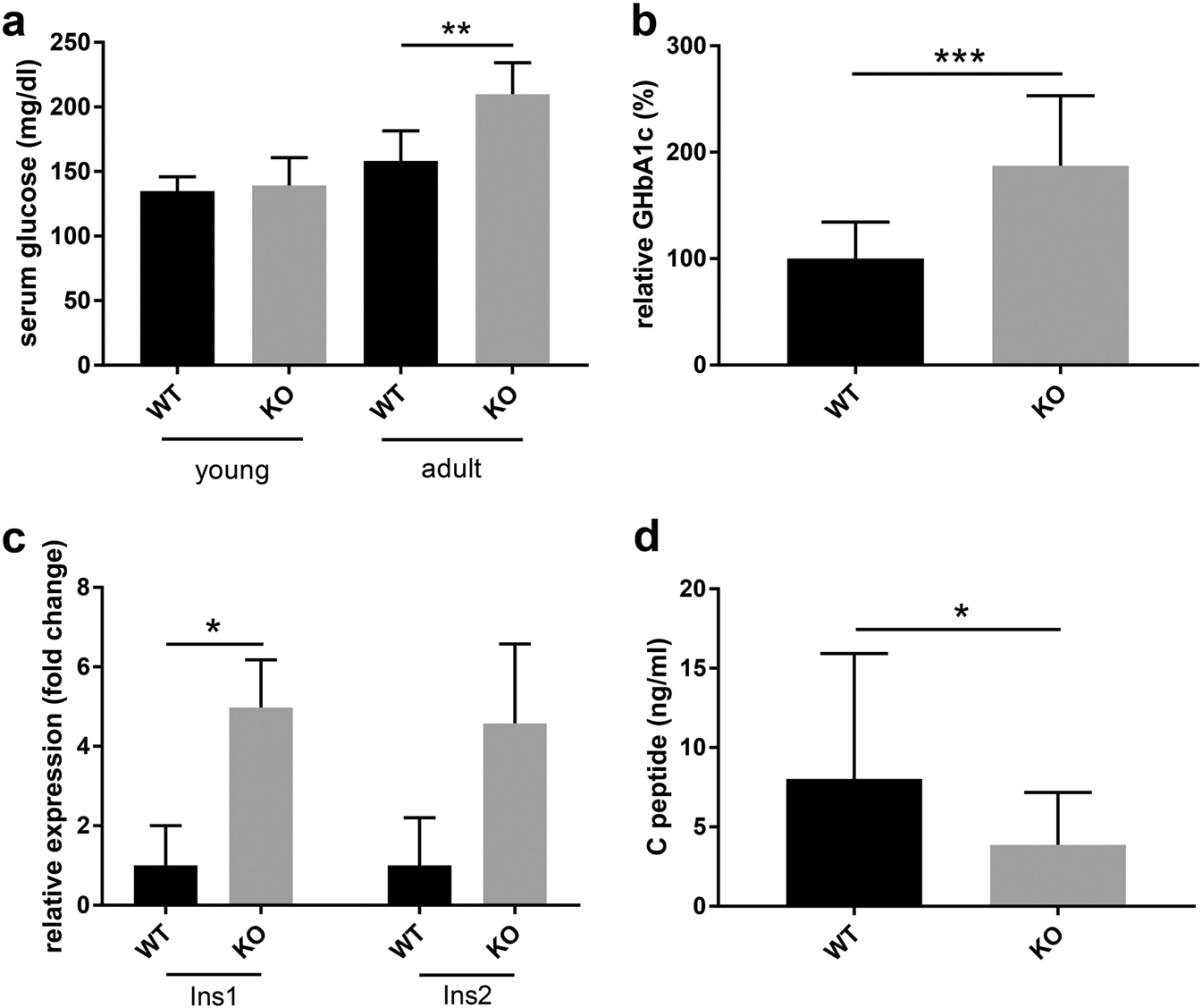
Exocrine pancreatic *Cosmc* KO induces diabetes in adult mice. **a** Measurement of fasting serum blood glucose in young animals (*n* = 10; *P* = 0.58) and adult mice (*n* = 15; *P* = 0.0005). **b** Quantification of HbA1c confirms diabetes in adult *Cosmc-*KO mice (*n* = 15 each; *P* < 0.0001). **c** Quantitative PCR analysis of Ins1 and Ins2 from total WT and *Cosmc-*KO pancreas mRNA (*n* = 3) indicates insulin overexpression by a factor of 5.0 (*P* = 0.025) and a factor of 4.58 (*P* = 0.053), respectively, in *Cosmc* KO. **d** Absolute C-peptide quantification in sera from WT (*n* = 18) and *Cosmc-*KO (*n* = 19) adult mice shows reduced C-peptide expression in *Cosmc-*KO mice (*P* = 0.043)

Having identified Cel as being critically involved in MODY8 pathogenesis as a major Tn-modified pancreatic protein, we analyzed glucose metabolism in WT and *Cosmc-*KO mice. First, fasting serum glucose was measured in a large cohort of young and adult mice, with significantly elevated glucose observed in aged *Cosmc-*KO and not in young mice (Fig. 4a). This result was further validated by the measurement of glycated hemoglobin (HbA1c), which was significantly elevated in adult mice, indicating long-term hyperglycemia (Fig. 4b). To further pinpoint the molecular mechanism of the observed diabetes, we assayed insulin (Ins1 and Ins2) expression levels in total pancreas derived from WT and *Cosmc-*KO animals. To our surprise, we found that both insulin genes were significantly upregulated in *Cosmc-*KO animals (Fig. 4c). As a key regulator of glucose metabolism, insulin is subjected to regulated proteolytic activation, resulting in the generation of insulin C-peptide. Thus, C- peptide level in the circulation reflects insulin activation. Additionally, ELISA-based quantification of C-peptide from sera of WT and *Cosmc-*KO mice revealed a significantly decrease in the latter (Fig. 4d).

**Fig. 5:**
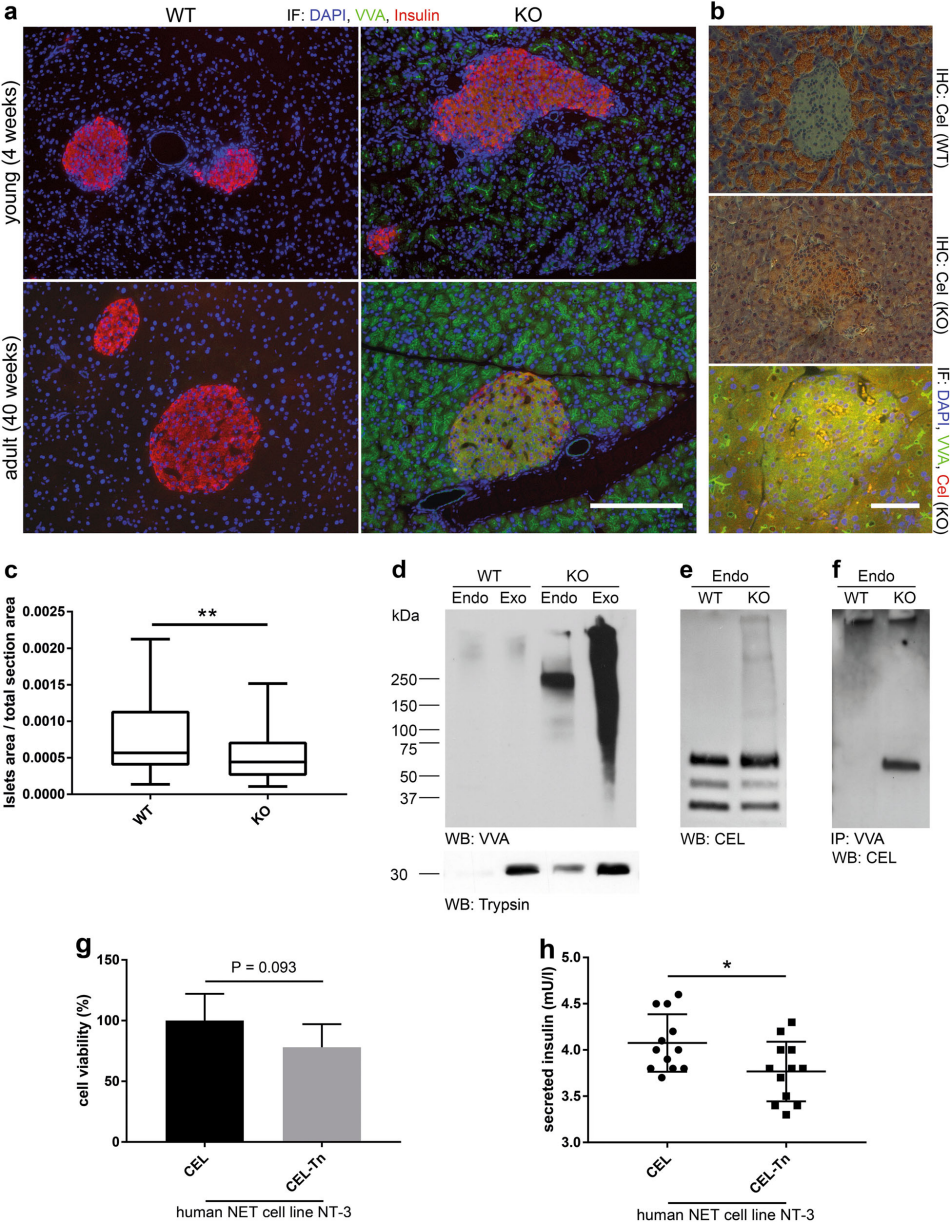
*Ptf1a*-Cre independent accumulation of Tn-modified proteins in pancreatic islets with consecutive beta cell failure. **a** Assessment of Tn antigen (green) and insulin expression (red) in pancreatic sections from 4 and 40-week-old WT and *Cosmc-*KO fasting mice. Tn antigen expression is only observed in *Cosmc-*KO tissue, and Tn antigen positivity is restricted to exocrine glands in young animals, whereas prolonged Tn antigen staining is observed in islets of Langerhans in aged mice. Scale bar equals 200 µm. **b** Cel IHC staining of adult WT and KO tissue and IF co-staining of VVA (green) and Cel (red). Scale bar equals 200 µm. **c** Quantification of islets of Langerhans of WT (*n* = 68) and KO (*n* = 64) pancreata (*P* = 0.004). **d** VVA Western blot analysis of endo- and exocrine pancreatic tissue derived from WT and *Cosmc-*KO animals. VVA positivity is specific for KO tissue, with most of the Tn-modified proteins found in exocrine tissue. Anti-trypsin was used as an exocrine marker. **e** Western blot analysis of WT and *Cosmc-*KO endocrine tissue using anti-Cel antibody. The analysis shows that the localization of Cel is not limited to the exocrine pancreas. **f** VVA precipitation from endocrine WT and KO tissue lysates with WB detection using anti-Cel antibody. **g** Comparison of NT-3 cell viability in the presence of 20 µM CEL and CEL-Tn after 48 h shows no significant difference. **h** CEL-Tn shows a significant negative regulation of glucose-stimulated insulin secretion in NT-3 cells. Cells were stimulated with 25 mM glucose for 60 min in the presence of 20 µM recombinant CEL or CEL-Tn

Next, we analyzed the expression of insulin/proinsulin and Tn antigen by immunohistochemistry in pancreata from fasting young and adult WT and *Cosmc-*KO mice. Consistent with previous reports, *Ptf1a*-Cre driven *Cosmc-*KO resulted in Tn antigen positivity restricted to pancreatic acinar cells in young animals (Fig. 5a). Insulin-producing beta cells were equally stained in WT and *Cosmc-*KO tissue in young mice. Interestingly, pronounced Tn antigen positivity was observed in islets of Langerhans in adult *Cosmc-*KO mice. To verify that the observed Tn positivity in the islets of Langerhans derived from exocrine proteins, we stained WT and KO tissue for Cel (Fig. 5b). Interestingly, KO tissue displayed marked Cel positivity compared to WT. This result was further substantiated by immunofluorescence co-staining of Tn and Cel in KO islets of Langerhans (Fig. 5b lower panel). Quantification of insulin-positive islet areas (islet area/total section area) revealed a significant reduction in KO pancreata (Fig. 5c). Analysis of Tn antigen expression revealed positivity and a distinct binding pattern in lysates derived from *Cosmc-*KO pancreatic islets. Anti-trypsin antibody was used to evaluate cross-contamination of exocrine-derived proteins in the endocrine compartment (Fig. 5d). It was apparent that elevated levels of Cel were found in islets of Langerhans derived from Cosmc-KO animals. This result is consistent with the staining presented in Fig. 5b. We next extracted genomic DNA from freshly isolated pancreatic islets and found no *Cosmc* deletion in endocrine cells, in contrast to exocrine tissue, by PCR (supplemental Figure 8). This result clearly demonstrates that Tn antigen positivity observed in pancreatic islets was not due to Cre-mediated *Cosmc* excision. We further analyzed cell lysates from isolated pancreatic islets and were able to detect Cel by Western blot in tissues derived from WT and *Cosmc-*KO animals. Cel, detected in lysates from *Cosmc-*KO endocrine pancreas, showed a characteristic staining pattern of additional high-molecular-weight bands compared to WT (Fig. 5e). In addition, we specifically detected Cel after VVA precipitation from lysates derived from *Cosmc-*KO pancreatic islets by Western blot (Fig. 5f). Lastly, we assayed the impact of CEL O-glycotype on cell viability and insulin secretion in vitro. We used human CEL recombinantly expressed in HEK293 WT and HEK293 simple cells (SC)^3^ to treat NT-3 cells^24^, an insulin-producing human neuroendocrine tumor cell line, in the presence of glucose. Comparative analysis of the impact of CEL-Tn and CEL on NT-3 cell viability displayed a trend after 48 h, wherein CEL-Tn reduced cell viability (Fig. 5g). Further, measurement of glucose-stimulated insulin secretion clearly indicated that CEL-Tn derived from HEK293 SC negatively regulated secretion compared to CEL with normal glycotype (Fig. 5h).

## Discussion

Cosmc is the essential chaperone for correct protein O-glycosylation, which in turn is essential for correct cellular function. Depletion of Cosmc results in embryonic lethality, highlighting its role in developmental processes^6^. Cosmc is ubiquitously expressed, but the biological functions of O- glycosylation have not been elucidated globally. The main reason for this is biological heterogeneity of cell types and tissues, leading to inconsistent phenotypes when aberrant O-glycosylation is induced by genetically switching off *Cosmc* or *T-synthase*. Recent examples are altered integrity of the glomerular filtration barrier, resulting in spontaneous proteinuria and rapidly progressing glomerulosclerosis^27^ and a compromised mucus layer, spontaneous microbe-dependent inflammation, and colitis^28^. Correct O-glycosylation in pancreatic acinar cells is required for normal zymogen granule formation and regulated protein secretion in vitro^29^. When O-linked glycosylation is perturbed, the secretory pathway is inhibited, accompanied by morphological alterations due to the aggregation of zymogen granules and association of aggregates with the trans-Golgi network membrane. Interestingly, DMBT1 has been identified as a structural zymogen granule protein involved in the regulation of the secretory pathway^30,31^. Prerequisite to secretion is functional protein folding in ER, mediated by multiple chaperoning systems. The 78-kDa glucose regulated protein (GRP78) is a general chaperone recognizing unfolded proteins by its hydrophobic residues^32^. GRP78 is O-glycosylated in many cancer cell lines, but GRP78 O-glycosylation is not linked to the unfolded protein response^3^. Interestingly, GRP78 associates with T-synthase in cancer cell lines deficient in Cosmc^33^. Generally, O-glycosylation confers resistance to proteolytic degradation, overall protein stability and regulated proteolysis^3,34^. Immature O-glycosylation thus affects regulated zymogen enzyme activation and presumably protein half-life, providing an explanation for the reduced activity of digestive enzymes observed in the present mouse strain.

We utilized the plant lectin VVA to enrich pancreatic Tn antigen glycoproteins that were subsequently analyzed by mass spectrometry. The approach to the identification of O-glycoproteins presented here differs from previous studies (in which genetically modified cell lines were used) by taking biological context and the physiological consequences of altered O-GalNAc glycosylation into account^3,12^. The O- glycoproteome has been only partially explored because conserved polypeptide motifs for the prediction of O-glycosylation sites are lacking^35^. Among all the glycan moieties identified as antigens so far, Tn and STn antigens have outstanding potential since both arise in a majority of human cancers^36–38^. Given that over one-half of all proteins encoded in the human genome are glycosylated^39^, it is not surprising that aberrant glycosylation may have an impact on diverse biological pathways.

Cel, previously described as O-glycosylated^15^, was found consistently among the identified Tn-modified glycoproteins here. Furthermore, our results indicate that the impairment of O-glycosylation on Cel was responsible for the observed MODY8-related phenotype, similar to mutation-induced C-terminal truncation^14^. So far, the pathophysiological mechanisms leading to the disease phenotype are poorly understood, although misfolding and aggregation of mutated *CEL* have been described to play a pivotal role in the pathogenic process^40^. Ectopically expressed and secreted C-terminal-truncated CEL can be endocytosed by human and rodent cell lines. Internalization of truncated CEL reduces the viability of pancreatic acinar and beta cell models, indicating the induction of apoptosis in pancreatic cells^41^. Our data suggest a pivotal role for the Cel C-terminal region, consisting of a VNTR including PEST sequences enriched in the amino acids proline, glutamate, serine, and threonine and clustered O-glycosylation sites in humans and mice^42^, which are important for normal intracellular protein processing^43^. Notably, the C-terminal domain of Cel harbors neither catalytic^44^ nor regulatory bile salt binding sites^45,46^, and mutation or complete truncation of the latter does not interfere with Cel enzyme activity^16^ Interestingly, the manifestation of MODY8 symptoms in patients is related to the number of VNTRs that include O-glycosylation sites^14^. Another report shows that a recombined allele of CEL and its pseudogene CELP confers susceptibility to chronic pancreatitis. The resulting Cel hybrid protein is characterized by a truncated VNTR region^47^.

In the mouse strain described here, Cel O-glycosylation remained immature, and diabetes onset was observed in mice older than 3 months of age, which is in agreement with previous reports on MODY8 as a progressive disease. The occurrence of pancreatic exocrine dysfunction in MODY8 patients is observed from infancy, and diabetes develops in the fifth decade of life^48^. Despite the fact that endocrine cells, especially insulin-producing beta cells, are not affected by *Cosmc* deletion in our mouse strain, we observed Tn positivity in adult mice. We further provide evidence for the presence of Cel-Tn in beta cells, causing impairment of insulin secretion and insulin maturation, resulting in reduced serum C-peptide serum in adult *Cosmc-*KO mice.

In accordance with the reported cluster of symptoms in MODY8 patients^14,49^, characterization of Cosmc-deficient mice revealed symptoms of exocrine pancreatic insufficiency with maldigestion, altered stool formation, impaired zymogen granule release and decreased enzymatic elastase and lipase activity. Moreover, *Cosmc-*KO mice did not show clinical signs of malnutrition after 8 weeks but had smaller pancreata than control animals. The enzyme activity levels tested were significantly lower, and total cholesterol, triglycerides and HDL decreased in blood samples, indicating severely reduced pancreatic acinar function. Fasting serum glucose and HbA1c were elevated, and fasting serum C-peptide was reduced, consistent with diabetes.

The mouse strain presented here with Cosmc deficiency and loss of core 1 glycans in pancreatic acinar cells showed a causal connection between differential O-glycosylation and exocrine pancreatic dysfunction, as well as diabetes, highlighting the relevance of O-glycosylation to the etiology and pathophysiology of human diseases^50–53^.

## Electronic supplementary material

Supplemental figures and figure legends

LC-MSMS-VVA_Cosmc_KO_supplement

## References

[1] Agre P (2016). Training the next generation of biomedical investigators in glycosciences. J. Clin. Invest..

[2] Hang HC, Bertozzi CR (2005). The chemistry and biology of mucin-type O-linked glycosylation. Bioorg. Med. Chem..

[3] Steentoft C (2013). Precision mapping of the human O-GalNAc glycoproteome through SimpleCell technology. EMBO J..

[4] Dahr W, Uhlenbruck G, Gunson HH, Van Der Hart M (1975). Molecular basis of Tn-polyagglutinability. Vox. Sang..

[5] Ju T, Brewer K, D’Souza A, Cummings RD, Canfield WM (2002). Cloning and expression of human core 1 beta1,3-galactosyltransferase. J. Biol. Chem..

[6] Wang Y (2010). Cosmc is an essential chaperone for correct protein O-glycosylation. Proc. Natl. Acad. Sci. USA.

[7] Sun Q, Ju T, Cummings RD (2011). The transmembrane domain of the molecular chaperone Cosmc directs its localization to the endoplasmic reticulum. J. Biol. Chem..

[8] Ohtsubo K, Marth JD (2006). Glycosylation in cellular mechanisms of health and disease. Cell.

[9] Obata J (2001). p48 subunit of mouse PTF1 binds to RBP-Jkappa/CBF-1, the intracellular mediator of Notch signalling, and is expressed in the neural tube of early stage embryos. Genes. Cells.

[10] Buchakjian MR, Kornbluth S (2010). The engine driving the ship: metabolic steering of cell proliferation and death. Nat. Rev. Mol. Cell Biol..

[11] Tollefsen SE, Kornfeld R (1983). The B4 lectin from Vicia villosa seeds interacts with N- acetylgalactosamine residues alpha-linked to serine or threonine residues in cell surface glycoproteins. J. Biol. Chem..

[12] Steentoft C (2011). Mining the O-glycoproteome using zinc-finger nuclease-glycoengineered SimpleCell lines. Nat. Methods.

[13] Johansson BB (2018). The role of the carboxyl ester lipase (CEL) gene in pancreatic disease. Pancreatology.

[14] Raeder H (2006). Mutations in the CEL VNTR cause a syndrome of diabetes and pancreatic exocrine dysfunction. Nat. Genet..

[15] Wang CS (1995). Isolation and characterization of human milk bile salt-activated lipase C-tail fragment. Biochemistry.

[16] Downs D, Xu YY, Tang J, Wang CS (1994). Proline-rich domain and glycosylation are not essential for the enzymic activity of bile salt-activated lipase. Kinetic studies of T-BAL, a truncated form of the enzyme, expressed in Escherichia coli. Biochemistry.

[17] Bruneau N, Nganga A, Fisher EA, Lombardo D (1997). O-Glycosylation of C-terminal tandem-repeated sequences regulates the secretion of rat pancreatic bile salt-dependent lipase. J. Biol. Chem..

[18] Neuman JC, Truchan NA, Joseph JW, Kimple ME (2014). A method for mouse pancreatic islet isolation and intracellular cAMP determination. J. Vis. Exp..

[19] Gout J., et al. Isolation and culture of mouse primary pancreatic acinar cells. *J. Vis. Exp.* **78** (2013). 10.3791/50514 PMC3855917 23979477

[20] Rindler M. J. Isolation of zymogen granules from rat pancreas. In: Bonifacino J. S., Basso M., Harford J. B., Lippincott-Schwartz J., Yamada K. M. (eds). *Current Protocols in Cell Biology*. John Wiley & Sons, Inc.: New York, 2006; Vol. **1**, pp 3.18.1–3.18.15. 10.1002/0471143030.cb0318s29 18228486

[21] Hofmann BT (2015). COSMC knockdown mediated aberrant O-glycosylation promotes oncogenic properties in pancreatic cancer. Mol. Cancer.

[22] Lutz D, Wolters-Eisfeld G, Schachner M, Kleene R (2014). Cathepsin E generates a sumoylated intracellular fragment of the cell adhesion molecule L1 to promote neuronal and Schwann cell migration as well as myelination. J. Neurochem..

[23] Wolters-Eisfeld G, Schumacher U (2017). Lectin histochemistry for metastasizing and non-metastasizing cancer cells. Methods Mol. Biol..

[24] Benten D (2018). Establishment of the first well-differentiated human pancreatic neuroendocrine tumor model. Mol. Cancer Res..

[25] Ju T (2011). A novel fluorescent assay for T-synthase activity. Glycobiology.

[26] Vembar SS, Brodsky JL (2008). One step at a time: endoplasmic reticulum-associated degradation. Nat. Rev. Mol. Cell Biol..

[27] Song K (2017). Loss of mucin-type O-glycans impairs the integrity of the glomerular filtration barrier in the mouse kidney. J. Biol. Chem..

[28] Kudelka MR (2016). Cosmc is an X-linked inflammatory bowel disease risk gene that spatially regulates gut microbiota and contributes to sex-specific risk. Proc. Natl. Acad. Sci. USA.

[29] De Lisle RC (2002). Role of sulfated O-linked glycoproteins in zymogen granule formation. J. Cell. Sci..

[30] De Lisle RC, Ziemer D (2000). Processing of pro-Muclin and divergent trafficking of its products to zymogen granules and the apical plasma membrane of pancreatic acinar cells. Eur. J. Cell Biol..

[31] De Lisle RC, Xu W, Roe BA, Ziemer D (2008). Effects of Muclin (Dmbt1) deficiency on the gastrointestinal system. Am. J. Physiol. Gastrointest..

[32] Lee AS (2005). The ER chaperone and signaling regulator GRP78/BiP as a monitor of endoplasmic reticulum stress. Methods.

[33] Ju T, Aryal RP, Stowell CJ, Cummings RD (2008). Regulation of protein O-glycosylation by the endoplasmic reticulum-localized molecular chaperone Cosmc. J. Cell. Biol..

[34] Rogers S, Wells R, Rechsteiner M (1986). Amino acid sequences common to rapidly degraded proteins: the PEST hypothesis. Science.

[35] Julenius K, Molgaard A, Gupta R, Brunak S (2005). Prediction, conservation analysis, and structural characterization of mammalian mucin-type O-glycosylation sites. Glycobiology.

[36] Kim GE (2002). Aberrant expression of MUC5AC and MUC6 gastric mucins and sialyl Tn antigen in intraepithelial neoplasms of the pancreas. Gastroenterology.

[37] Springer GFT (1984). and Tn, general carcinoma autoantigens. Science.

[38] Terada T, Nakanuma Y (1996). Expression of mucin carbohydrate antigens (T, Tn and sialyl Tn) and MUC-1 gene product in intraductal papillary-mucinous neoplasm of the pancreas. Am. J. Clin. Pathol..

[39] Zhang H (2010). Simultaneous characterization of glyco- and phosphoproteomes of mouse brain membrane proteome with electrostatic repulsion hydrophilic interaction chromatography. Mol. Cell. Proteom..

[40] Johansson BB (2011). Diabetes and pancreatic exocrine dysfunction due to mutations in the carboxyl ester lipase gene-maturity onset diabetes of the young (CEL-MODY): a protein misfolding disease. J. Biol. Chem..

[41] Torsvik J (2014). Endocytosis of secreted carboxyl ester lipase in a syndrome of diabetes and pancreatic exocrine dysfunction. J. Biol. Chem..

[42] Higuchi S, Nakamura Y, Saito S (2002). Characterization of a VNTR polymorphism in the coding region of the CEL gene. J. Hum. Genet..

[43] Lombardo D (2001). Bile salt-dependent lipase: its pathophysiological implications. Biochim. Biophys. Acta.

[44] DiPersio LP, Fontaine RN, Hui DY (1990). Identification of the active site serine in pancreatic cholesterol esterase by chemical modification and site-specific mutagenesis. J. Biol. Chem..

[45] Aubert E, Sbarra V, Le Petit-Thevenin J, Valette A, Lombardo D (2002). Site-directed mutagenesis of the basic N-terminal cluster of pancreatic bile salt-dependent lipase. Functional significance. J. Biol. Chem..

[46] Aubert-Jousset E, Sbarra V, Lombardo D (2004). Site-directed mutagenesis of the distal basic cluster of pancreatic bile salt-dependent lipase. J. Biol. Chem..

[47] Fjeld K (2015). A recombined allele of the lipase gene CEL and its pseudogene CELP confers susceptibility to chronic pancreatitis. Nat. Genet..

[48] Raeder H (2014). Carboxyl-ester lipase maturity- onset diabetes of the young is associated with development of pancreatic cysts and upregulated MAPK signaling in secretin-stimulated duodenal fluid. Diabetes.

[49] Tjora E (2013). Severe pancreatic dysfunction but compensated nutritional status in monogenic pancreatic disease caused by carboxyl-ester lipase mutations. Pancreas.

[50] Fu J (2011). Loss of intestinal core 1-derived O- glycans causes spontaneous colitis in mice. J. Clin. Invest..

[51] Ju T, Cummings RD (2005). Protein glycosylation: chaperone mutation in Tn syndrome. Nature.

[52] Wang Y (2012). Platelet biogenesis and functions require correct protein O-glycosylation. Proc. Natl. Acad. Sci. USA.

[53] Bjorkqvist J (2015). Defective glycosylation of coagulation factor XII underlies hereditary angioedema type III. J. Clin. Invest..

