# Supplementary material for: Loss of complex O-glycosylation impairs exocrine pancreatic function and induces MODY8-like diabetes in mice
Source: Exp Mol Med. 2018 Oct 10;50(10):133. doi: 10.1038/s12276-018-0157-3 (PMC6180059; doi:10.1038/s12276-018-0157-3)
Supplement: Supplementary file 1 — Supplemental figures and figure legends [file 12276_2018_157_MOESM1_ESM.pdf]

Supplemental figures and figure legends

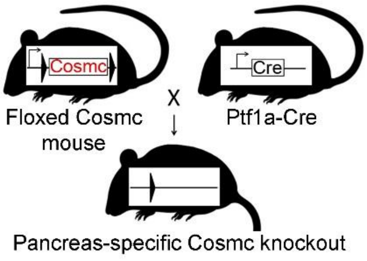

Supplemental Figure 1. Scheme of mouse model with conditional *Cosmc* deficiency in the pancreas. Homozygously floxed *Cosmc*$^{flox/flox}$ females were interbred with the Cre-deleter mouse strain *Ptf1a*$^{+/Cre}$ to generate *Ptf1a*$^{+/Cre}$;*Cosmc*$^{flox/y}$ male offspring.

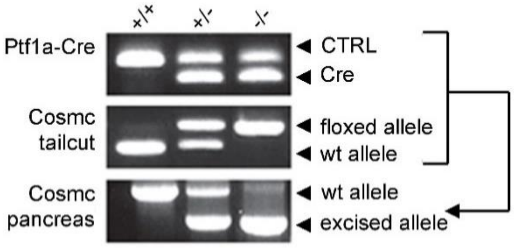

Supplemental Figure 2. Representative agarose-gels depicting genotyping strategy for Cre, *Cosmc* and *Cosmc* allele excision in hetero- and homozygous animals.

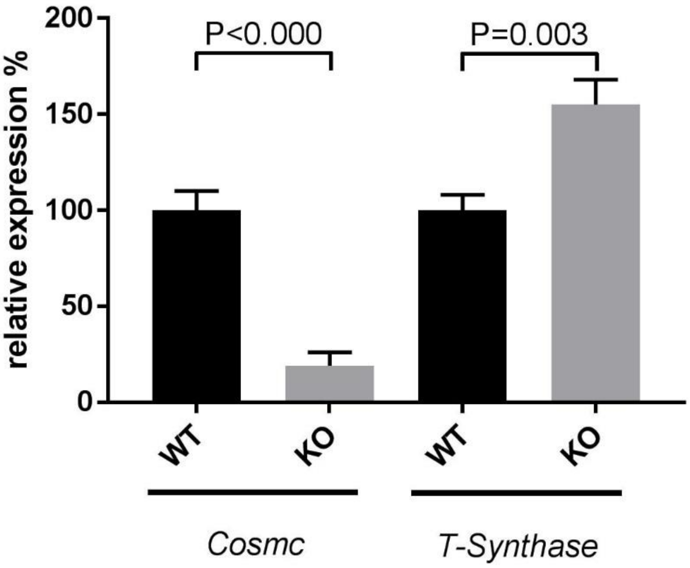

Supplemental Figure 3. Expression analyses of *Cosmc* and *T-Synthase* confirmed *Cosmc* deficiency using qRT-PCR. Of note, we found *T-Synthase* overexpressed in *Cosmc* KO pancreatic tissue (n = 4). Unpaired two-tailed Student’s t-test was used to test the level of significance.

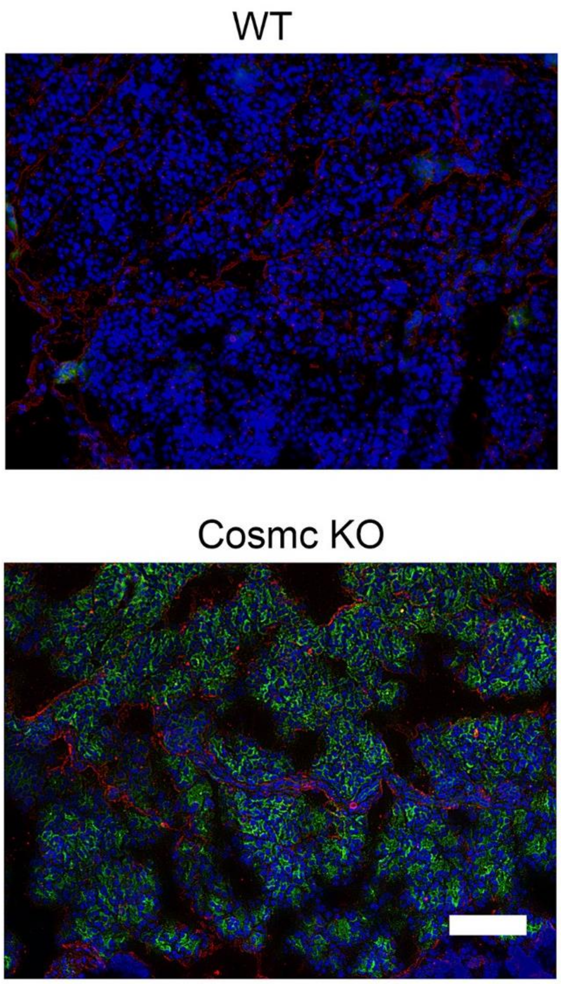

Supplemental Figure 4. Immunofluorescence using fluorescein-conjugated VVA was used to visualize Tn expression in *Cosmc* KO tissue (green). Endothelial cells were stained red using biotinylated *Sambucus nigra* lectin (SNA, EBL) complexed with NeutrAvidin 594. DAPI was used to counterstain nuclei. The scale bar equals 50 µm.

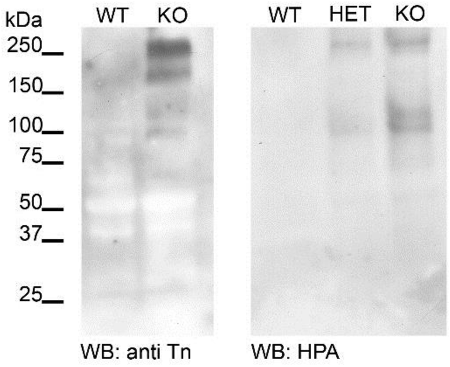

Supplemental Figure 5. Western and Far-Western blots of WT and KO pancreas lysates using an anti-Tn antigen antibody and the snail lectin *Helix pomatia agglutinin* (HPA) for specific detection of O-GalNAc modified proteins. Reduced levels of Tn proteins are detectable in tissue derived from heterozygous Cosmc KO (HET).

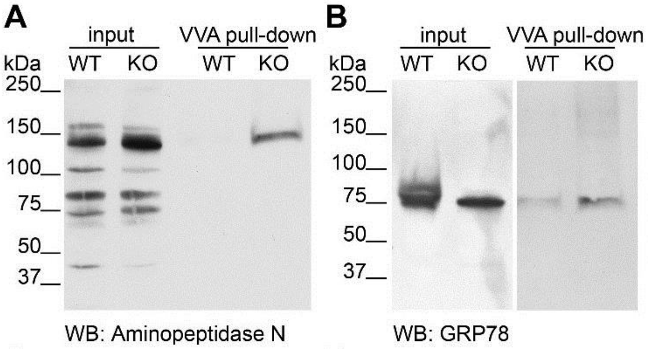

Supplemental Figure 6. WB of VVA pull-downs from WT and *Cosmc* KO pancreatic lysates using specific antibodies directed against (A) aminopeptidase N and (B) GRP78 for detection. Target proteins were specifically detected in pull-downs from *Cosmc* KO pancreatic tissue corresponding to molecular weight in input.

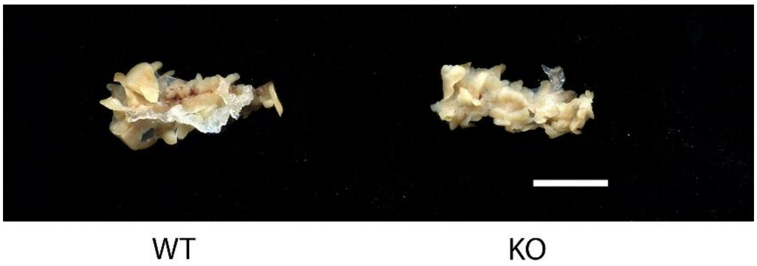

Supplemental Figure 7. Representative images of pancreata derived from aged mice. Scale bar equals 1 cm.

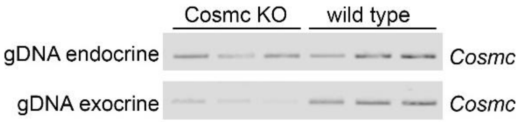

Supplemental Figure 8. Analytical agarose gels of RT-PCRs depicting status of genomic *Cosmc* in gDNA derived from exocrine and endocrine KO and WT (n=3) pancreata. *Cosmc* is specifically excised in exocrine KO tissue (no or faint band).
